# Rehabilitation services in disaster response

**DOI:** 10.2471/BLT.15.157024

**Published:** 2017-02-01

**Authors:** Jody-Anne Mills, Jo Durham, Venkatakannan Packirisamy

**Affiliations:** a School of Public Health, Herston Road, University of Queensland, Herston, Brisbane, Queensland, 4006, Australia.; b International Committee of the Red Cross, Juba, South Sudan.

The United Nations *Convention on the Rights of Persons with Disabilities* has firmly placed disability in a human rights perspective. In the context of the Convention, disability refers to activity limitations and participation restrictions that result from the interaction of an impairment of body function or structure with environmental factors. The Convention addresses the rights of persons with disability in all aspects of life, including education and community integration and their right to equal access to services, such as schools, medical facilities and communication. Articles 25 and 26 explicitly state the right to health and access to health-related rehabilitation for persons with disabilities,^1^ in all settings and situations, including during emergencies and natural disasters. Adherence to the Convention however, can be particularly challenging in a disaster response given the different economic and social vulnerabilities including poverty, as well as environmental hazards and damaged infrastructure. This can be further compounded by the limited time emergency medical teams are typically deployed for as part of a disaster response.^2^^,^^3^

Often when disasters occur in low- and middle-income countries, there is an influx of specialist health workers from high-income countries. In affected countries where health systems are weak, and health-related rehabilitation services are not a priority, this influx of specialist health workers can result in individuals with certain types of traumatic injuries – such as spinal cord injuries, amputations, brain injuries or peripheral nerve injuries – surviving with long-term impairment and associated disability, in an environment with limited capacity to address their ongoing needs.^4^ Until recently, rehabilitation professionals have been underrepresented in emergency medical teams. In recent years however, guidance documents have started to promote the importance of inclusion and/or close coordination of rehabilitation professionals with these teams.^5^^,^^6^ Inclusion of rehabilitation personnel in emergency medical teams indicates a trend towards patient-centred care and offers timely access to rehabilitation intervention to those who have sustained injuries, which has been found to prevent complications, speed recovery and optimize functional outcomes for the injured.^2^^,^^3^ Rehabilitation personnel in emergency medical teams are also well placed to provide mentorship and support local staff to manage the surge of individuals with traumatic injuries and the ongoing care needs of those affected.

## A multidisciplinary response

Historically, a medical model –where a person's impairment is perceived to be the problem, and the focus is on fixing the problem – has dominated emergency medical teams. However, the Convention with its focus on a social model of disability, in which disability is seen as a function of the interactions between an impairment and the barriers in the environment, implies the need to provide a patient-centred, multidisciplinary model of care in hospital settings. Article 26 in particular implies the need to incorporate rehabilitation professionals into staffing arrangements starting from the acute care phase. Having a multidisciplinary team and an effective coordination mechanism allows emergency medical teams to address the needs of their patients holistically and link appropriately with other service providers, including specialists, facilities and disability-orientated nongovernmental organizations. In light of the surge in traumatic injury seen in disaster events, it is crucial that emergency medical teams assess their capacity and prepare themselves to coordinate with both local and international agencies. Such coordination will allow for patient care to continue beyond the medical teams’ departure and better preparation of the injured for social and economic inclusion.

The classification and minimum standards for foreign medical teams in sudden onset disaster^7^ acknowledges the need for multidisciplinary management of disabling injuries. Previous publications, such as the 2011 Humanitarian Action Summit Surgical Working Group consensus statements regarding the multidisciplinary care of limb amputation,^6^ also highlighted the critical need for surgical providers to have at least a basic understanding of rehabilitative principles and to ensure that decisions in the acute stages of care consider the patient’s needs beyond the operating theatre. However, little to no guidance has so far been available to emergency medical teams for staffing, training or equipment requirements for rehabilitation. In addition, those documents that do encourage the inclusion of rehabilitation in disaster response are relatively novel and their success in improving standards of service delivery are yet to be practically tested.^7^

## Operationalizing the response

Disaster response is often implemented in three phases; (i) provision of emergency services and public assistance, with emphasis on life-saving (immediate response), (ii) provision of services in the immediate aftermath of a disaster, with the aim to restore the affected area to pre-crisis conditions (recovery), (iii) improvement of conditions in the affected areas relative to pre-crisis conditions (development). This means capacity building in the field of rehabilitation requires a certain level of flexibility when implemented in the transitions between these different phases. Traditionally, the immediate response and recovery phases have occurred as distinct phases of the response intervention to that of development. The length of the response and recovery phases varies because of different socioeconomic and political contextual factors, the type of disaster and the capacity of the relevant authorities.^8^

Given the surge of impairment and associated disability among affected populations seen in disasters, and the ongoing nature of the Convention’s implementation in different countries, there is a need for effective transitions and coordination from disaster response to recovery and coordination between agencies in these phases. To this effect, the preparation of local health and social services is paramount to seeing the recovery and continuing health and health well-being of those who sustain disabling injuries. International organizations with a mandate to establish a long-term presence in a country affected by a disaster are best placed to promote local capacity building efforts for disaster response and development. While the time-restricted nature of emergency medical teams’ intervention may limit their ability to provide long-term capacity-building support, their presence in the country during disaster response presents an opportunity for skill transfer and mentoring. Inter-agency coordination will play a vital role in integrating the initiatives made by foreign teams deployed for a short or long time into the local disability and rehabilitation services.

There are clear differences in the skill sets required for the response and development phases. However, blurring the lines between these two phases has complex implications for planning and funding, and demands a shift away from traditional perspectives. The extent to which relief organizations are equipped to provide long-term capacity building and integrate into development strategies merits further research.^9^

## Monitoring and evaluation

Coordination between different agencies in the transition from response to recovery and development would facilitate effective monitoring and evaluation related to rehabilitation and disability. In accordance with Article 31 of the Convention, States Parties are obligated to collect statistical and research data.^1^ For such data to be meaningful, it should measure both short-term functional outcomes, such as mobility, pain or discomfort, and longer-term outcomes related to performance and participation. However, the scope of stakeholders engaged in disaster response and their information need is diverse. To make the data comparable across agencies, a certain level of consistency is required in the type of data collected and the data collection methods and evaluation by various agencies involved in the response and development phase. Even where the institutional, legal, and organizational structures for the collection of disability-related health information exist, in low-resource settings the functional capability of these structures is often weak, limiting the availability and usability of the data. Having an on-going injury surveillance system, developed based on an international standard, and that can continue to operate in disaster response situations will help facilitate the data collection process. The definition and use of outcome indicators will ensure that comparable longer-term functional outcomes are captured. In the short term, establishing minimum standards for data collection – which includes information on mobility, functional outcomes, and where possible, environmental and social factors, and culturally appropriate measures of activities of daily living – will be a good start in meeting the needs of people with disabilities.

While the need to improve data collection in humanitarian response is clear, concerns exist regarding how it is prioritized, especially for emergency medical teams. Quality data collection can add demands on service providers and often requires additional human and financial resources. For this reason, it is imperative that data-collection systems are pre-established, organized and coordinated, to ensure optimal usability and feasibility in response scenarios. In addition, the benefits of quality monitoring and evaluation efforts need to be demonstrated to encourage donors and service providers to invest in and develop necessary resources. While the need for monitoring in humanitarian response is being reflected in best practice guidance for disaster response, the lack of detail contained in current guidance limits their ability to effect real change and to be effective. More technical and operational guidance on monitoring is required for emergency medical teams and affected countries.

Protecting and promoting the rights of the vulnerable is at the core of disaster response. However, there is far to go before the rights of persons with disabilities are recognized to the same extent as their able-bodied counterparts. There remains an ongoing need to promote a rights-based model of disability at both a clinical and political level, with adequate acknowledgement of the needs of those with newly acquired and pre-existing disability in the early stages of care. Linkages and collaboration with disabled people’s organizations and disability nongovernmental organizations in the affected area will strengthen disability-inclusion in disaster response and support the transition of those with disability into the community. Ultimately, a more patient-centred and holistic model of care in the acute hospital setting will ensure optimal functional outcomes and better prepare people for social and economic inclusion.

At the minimum, protection and promotion of the rights of persons with disabilities will require the following five actions by national governments: (i) establishing inter-agency coordination and developing linkages with disabled people’s organizations; (ii) developing protocols for handover to rehabilitation and appropriate follow-up support; (iii) establishing effective monitoring and evaluation; (iv) supporting capacity development, including training in the World Health Organization’s forthcoming minimum standards on rehabilitation for emergency medical teams; (v) increasing awareness of obligations and duties under the *Convention on the Rights of Persons with Disabilities.*
